# Effects of Different Sera Conditions on Olfactory Ensheathing Cells *in Vitro*

**DOI:** 10.3390/ijms16010420

**Published:** 2014-12-26

**Authors:** Meng Lu, Jun Dong, Teng Lu, Hongjun Lv, Pinglin Yang, Zhijian Cheng, Jin Li, Baobao Liang, Junkui Xu, Haopeng Li, Xijing He

**Affiliations:** 1 Second Department of Orthopedics, Second Affiliated Hospital of Xi’an Jiaotong University, 157 West Five Road, Xincheng District, Xi’an 710004, Shaanxi, China; E-Mails: lumeng6020@163.com (M.L.); dongjun@stu.xjtu.edu.cn (J.D.); luteng@stu.xjtu.edu.cn (T.L.); yplyplypl1974@163.com (P.Y.); czj198506@gmail.com (Z.C.); jin_lee1218@163.com (J.L.); LHP-3993@163.com (H.L.); 2 Department of Endocrinology, First Affiliated Hospital of Xi’an Jiaotong University, Yanta West Road, No. 277, Xi’an 710061, China; E-Mail: njdxbjdx@163.com; 3 Department of Plastic Surgery, Second Affiliated Hospital of Xi’an Jiaotong University, West Five Road, No. 157, Xi’an 710004, China; E-Mail: xuebaocc@stu.xjtu.edu.cn; 4 Department of Orthopedics, Xi’an Honghui hospital of Xi’an Jiaotong University, Nanguo Road, No. 76, Xi’an 710054, China; E-Mail: xujunkui0304@stu.xjtu.edu.cn

**Keywords:** rat serum, fetal bovine serum, olfactory ensheathing cells, concentration, culture

## Abstract

Transplantation of olfactory ensheathing cells (OEC) is a promising therapy in spinal cord injury (SCI) treatment. However, the therapeutic efficacy of this method is unstable due to unknown reasons. Considering the alterations in the culture environment that occur during OEC preparation for transplantation, we hypothesize that these changes may cause variations in the curative effects of this method. In this study, we compared OEC cultured in medium containing different types and concentrations of serum. After purification and passage, the OEC were cultured for 7 days in different media containing 5%, 10%, 15% or 20% fetal bovine serum (FBS) or rat serum (RS), or the cells were cultured in FBS-containing medium first, followed by medium containing RS. In another group, the OEC were first cultured in 10% FBS for 3 days and then cultured with rat spinal cord explants with 10% RS for another 4 days. An MTT assay and P75 neurotrophin receptor immunofluorescence staining were used to examine cell viability and OEC numbers, respectively. The concentration of neurotrophin-3 (NT-3), which is secreted by OEC into the culture supernatant, was detected using the enzyme-linked immunosorbent assay (ELISA). RT-PCR was applied to investigate the NT-3 gene expression in OEC according to different groups. Compared with FBS, RS reduced OEC proliferation in relation to OEC counts (χ^2^ = 166.279, df = 1, *p* < 0.01), the optical density (OD) value in the MTT assay (χ^2^ = 34.730, df = 1, *p* < 0.01), and NT-3 concentration in the supernatant (χ^2^ = 242.997, df = 1, *p* < 0.01). OEC cultured with spinal cord explants secreted less NT-3 than OEC cultured alone (*F* = 9.611, df = 5.139, *p* < 0.01). Meanwhile, the order of application of different sera was not influential. There was statistically significant difference in NT-3 gene expression among different groups when the serum concentration was 15% (χ^2^ = 64.347, df = 1, *p* < 0.01). In conclusion, different serum conditions may be responsible for the variations in OEC proliferation and function.

## 1. Introduction

Olfactory ensheathing cells (OEC) are glial cells that have the ability to switch from a non-myelinating to a myelinating state [1]. These cells support neuronal regeneration both within the olfactory system and elsewhere in the central nervous system [1]. Animal experiments verified that the physiological changes in transplanted OEC primarily include the following two aspects: remyelination and the production of neurotrophic factors [1,2,3,4]. Currently, the safety of OEC in the treatment of spinal cord injury (SCI) patients has been demonstrated in phase I clinical trials [5,6]. However, OEC transplantation in the treatment of these patients remains controversial because the effects appear to be variable [7,8]. Specifically, neurological improvement in most patients was not apparent after a long-term clinical follow-up [6,9]. OEC from olfactory bulbs or olfactory mucosa are primarily cultured with medium that contains fetal bovine serum [10,11], and the environment for the survival of the OEC changes from the previous fetal bovine serum-containing medium into the internal environment of the body after transplantation. Previous research has evaluated limb motor function improvement and axonal regeneration and remyelination under the microscope [12,13,14], and a few studies have investigated the secretion of neurotrophic factors after transplantation. To determine whether drastic changes in the environment affect the growth status of transplanted OEC, we conducted a series of experiments to observe the survival state and function of OEC cultured in media with different types and concentrations of serum.

## 2. Results

### 2.1. Immunofluorescence Results and OEC Counts

**Figure 1:**
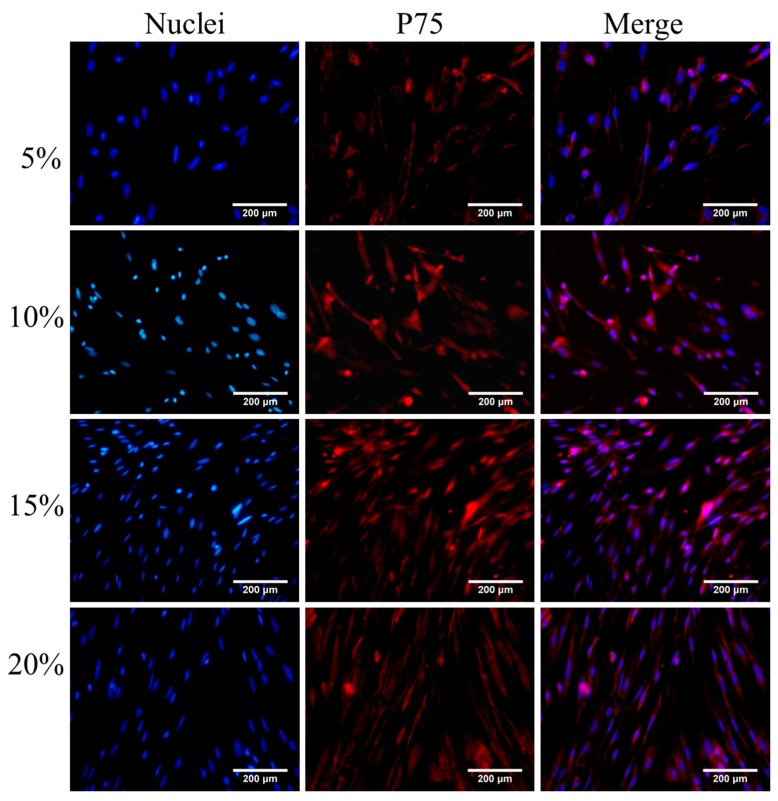
Immunofluorescence staining of olfactory ensheathing cells (OEC) cultured in DF-12 medium containing fetal bovine serum (FBS) (concentrations: 5%, 10%, 15%, 20%) for 7 days. The concentrations of FBS are listed on the left. OEC nuclei stained with DAPI are presented in the left column, P75 of OEC stained with Cy3 are presented in the middle column. The right column is the merged nuclei and P75. Scale bar is 200 μm.

**Figure 2:**
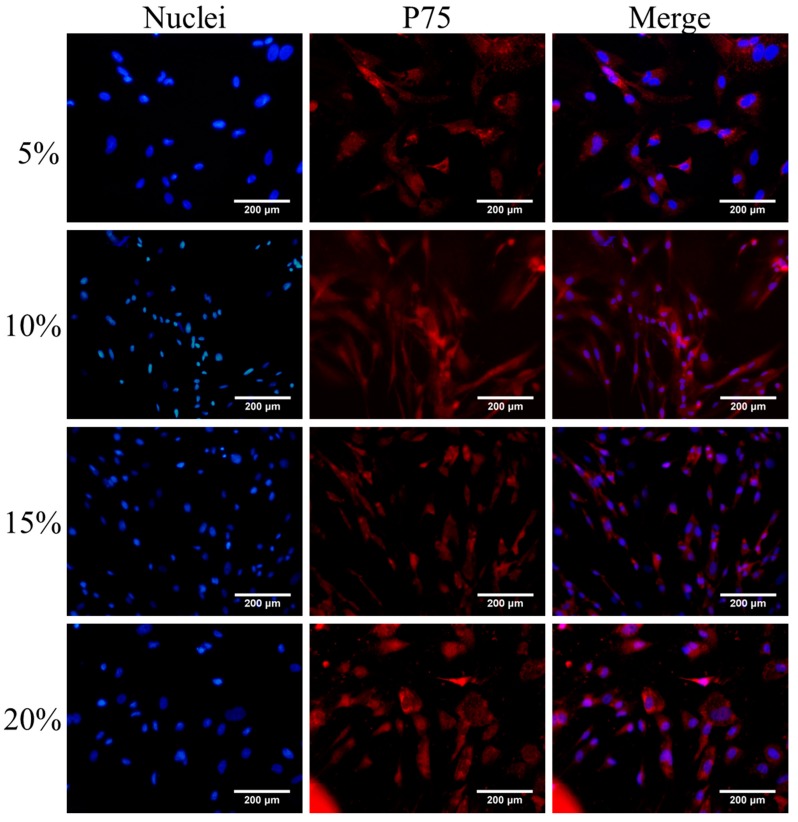
Immunofluorescence staining of OEC cultured in DF-12 medium containing rat serum (RS) (concentrations: 5%, 10%, 15%, 20%) for 7 days. The concentrations of RS are listed on the left. OEC nuclei stained with DAPI are presented in the left column, P75 of OEC stained with Cy3 are presented in the middle column, and the right column is the merged nuclei and P75. Scale bar is 200 μm.

**Figure 3:**
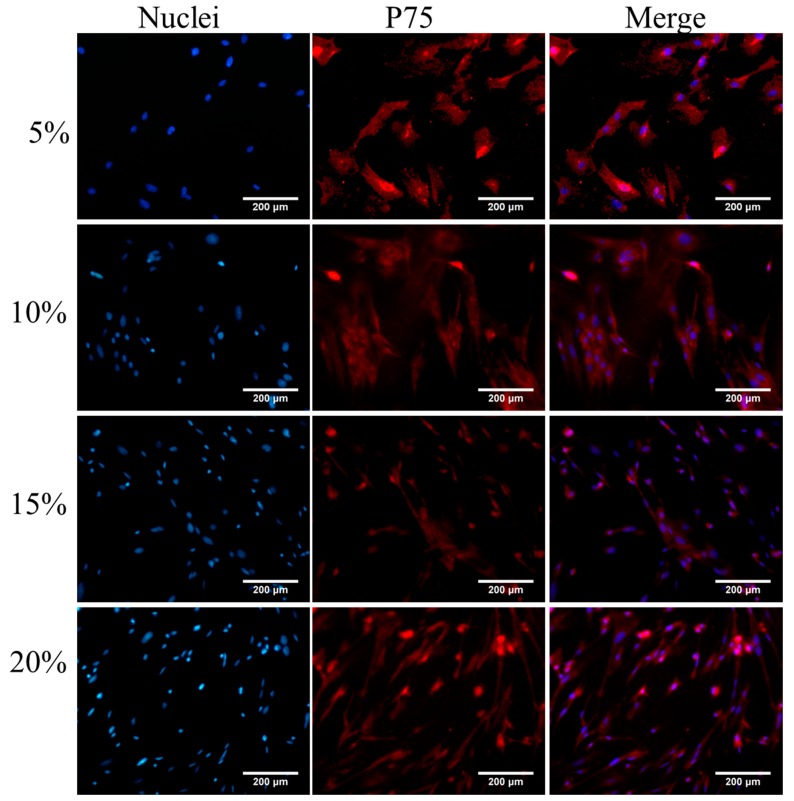
Immunofluorescence staining of OEC first cultured in DF-12 medium containing 10% FBS for 3 days and then cultured in DF-12 medium containing different concentrations (5%, 10%, 15%, 20%) of RS for 4 days. The different concentrations of RS are listed on the left. OEC nuclei stained with DAPI are presented in the left column, P75 of OEC stained with Cy3 are presented in the middle column, and the right column is the merged nuclei and P75. Scale bar is 200 μm.

**Figure 4:**
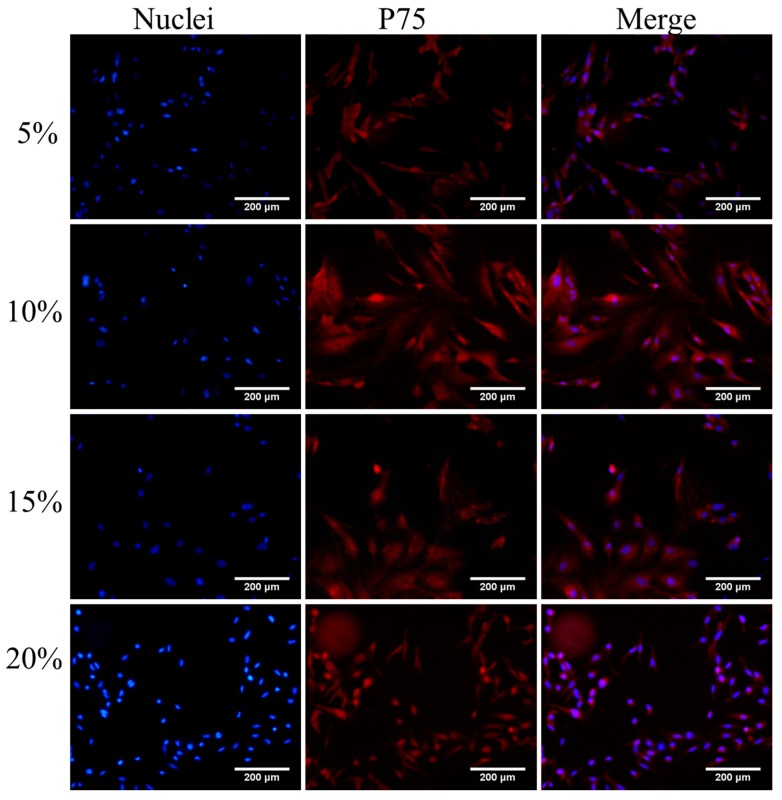
Immunofluorescence staining of OEC first cultured in DF-12 medium containing FBS (concentrations: 5%, 10%, 15%, 20%) for 3 days and then cultured in DF-12 medium containing RS (concentrations: 5%, 10%, 15%, 20%) for 4 days. The different concentrations of serum are listed on the left, and the concentrations of FBS and RS in one group were the same. OEC nuclei stained with DAPI are presented in the left column, P75 of OEC stained with Cy3 are presented in the middle column, and the right column is the merged nuclei and P75. Scale bar is 200 μm.

**Figure 5:**
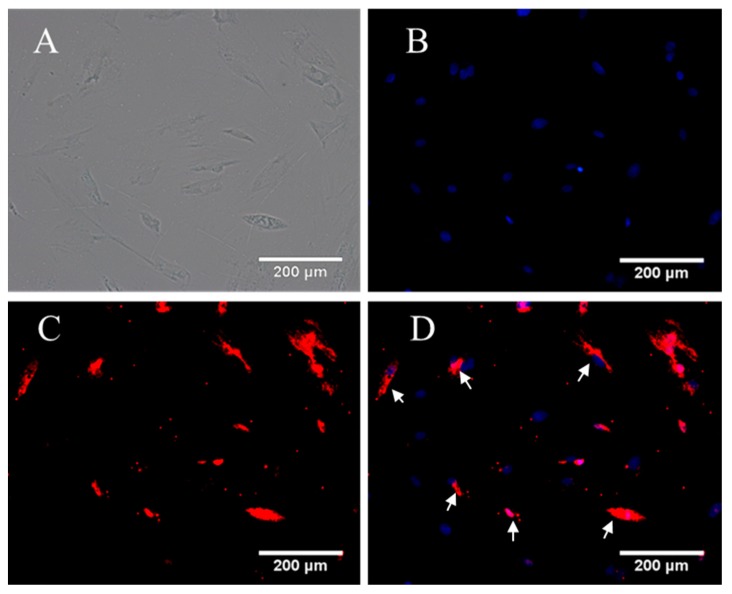
Immunofluorescence staining of OEC first cultured in DF-12 medium containing 10% FBS for 3 days and then cultured in DF-12 medium containing 10% RS and spinal cord explants for 4 days. Many cells were cultured; however, only a few OEC survived (white arrow). (**A**) OEC under an optical microscope; (**B**) OEC nuclei stained with DAPI; (**C**) OEC stained with Cy3; and (**D**) the merged B and C. Scale bar is 200μm.

**Figure 6:**
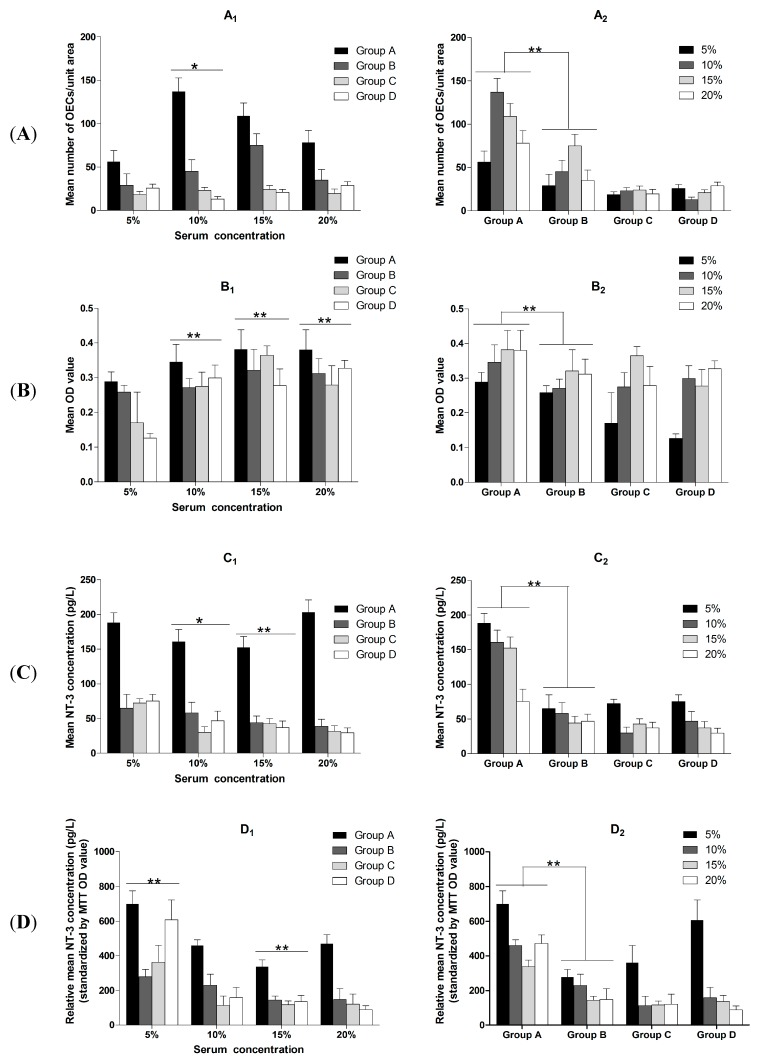
MTT assay and P75 neurotrophin receptor immunofluorescence staining were used to examine cell viability and OEC numbers, respectively. The concentration of NT-3 in the culture supernatant was detected by ELISA. Results which are analyzed according to different culture process at the same serum concentration are shown in **A_1_**, **B_1_**, **C_1_** and **D_1_**. Results analyzed according to different serum concentrations when culture process is the same are shown in **A_2_**, **B_2_**, **C_2_** and **D_2_**. The different serum concentrations are 5%, 10%, 15% and 20%. Group A, after trypsinization, OEC were placed onto slides and cultured in different concentrations (5%, 10%, 15%, 20%) of FBS for 7 days; Group B, after trypsinization, OEC were cultured in different concentrations (5%, 10%, 15%, 20%) of RS for 7 days; Group C, after trypsinization, OEC were cultured in 10% FBS for 3 days and then cultured in four different concentrations (5%, 10%, 15%, 20%) of RS for another 4 days; and Group D, after trypsinization, OEC were placed onto slides, cultured in four different concentrations (5%, 10%, 15%, 20%) of FBS for 3 days, and then cultured in RS (concentration: 5%, 10%, 15%, 20%) for another 4 days. (**A**) the mean number of OEC per unit area was counted. There was statistically significant difference when comparing 10% serum with 5% serum. (**A_1_**). A statistically significant difference of the mean number of OEC between Group A (FBS) and Group B (RS) was also observed (**A_2_**); (**B**) MTT assay was used to investigate the cell viability of OEC. There was statistically significant difference when comparing the serum concentration of 10%, 15% and 20% with serum concentration of 5% respectively (**B_1_**). There was statistically significant difference of the mean OD value between Group A (FBS) and Group B (RS) (**B_2_**); (**C**) the concentration of NT-3 in the culture supernatant was detected by ELISA. Comparing with serum concentration of 5%, there was statistically significant difference when the serum concentration was 10% or 15% (**C_1_**). There was statistically significant difference between Group A (FBS) and Group B (RS) (**C_2_**); (**D**) Relative mean NT-3 concentration was standardized by OD values of MTT. A statistically significant difference was detected when comparing the serum concentration of 5% with serum concentration of 10%. The significant difference was also detected when comparing the serum concentration of 15% with concentration of 20% (**D_1_**). There was statistically significant difference between Group A (FBS) and Group B (RS) (**D_2_**). The data was presented as mean ± SD. One asterisk means that there is statistically significant difference and *p* < 0.05. Two asterisks mean that there is statistically significant difference and *p* < 0.01 (Supplemental Table S1–S3).

**Figure 7:**
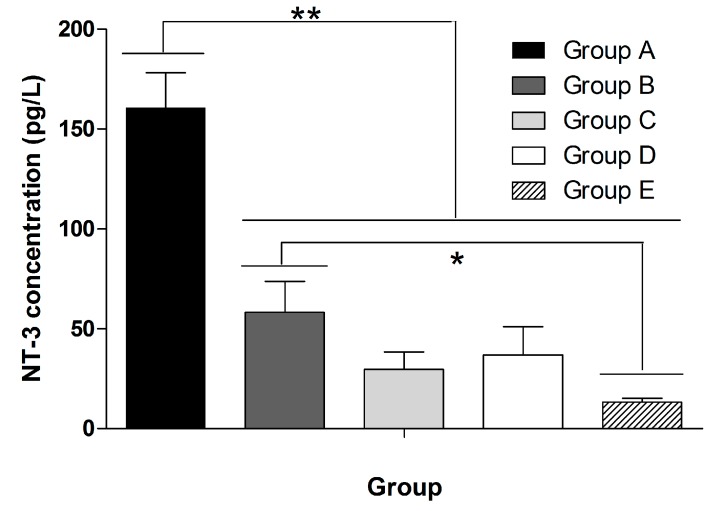
The concentration of NT-3 in the culture supernatant was detected by ELISA. Group A, after trypsinization, OEC were placed onto slides and cultured in 10% FBS for 7 days; Group B, after trypsinization, OEC were cultured in RS (concentration:10%) for 7 days; Group C, after trypsinization, OEC were cultured in 10% FBS for 3 days and then cultured in 10% RS for another 4 days; and Group D, after trypsinization, OEC were placed onto slides, cultured in 10% FBS for 3 days, and then cultured in 10% RS for another 4 days. Group E, the OEC were first cultured in 10% FBS for 3 days and then cultured with rat spinal cord explants with 10% RS for another 4 days. The data was presented as mean ± SD. There was statistically significant difference between Group A and Group B, Group A and Group C, Group A and Group E. Significant difference was also detected between Group B and Group E. The data was presented as mean ± SD. One asterisk means that there is statistically significant difference and *p* < 0.05. Two asterisks mean that there is statistically significant difference and *p* < 0.01.

After P75 immunofluorescence staining, an increased number of cells in the visual field were observed (Figure 1, Figure 2, Figure 3 and Figure 4). The immunofluorescence of OEC from Group E was shown in Figure 5. The number of OEC per unit area was shown in Figure 6A_1_,A_2_. A significant difference was detected when comparing the serum concentration at 10% with the concentration at 5% (χ^2^ = 3.875, df = 1, *p* = 0.049, <0.05) (Figure 6A_1_). The number of OEC in Group A was greater than the number in the Group B, and the difference was statistically significant (χ^2^ = 166.279, df = 1, *p* < 0.01) (Figure 6A_2_). No statistically significant difference was observed regarding the different orders in which the serum was applied (χ^2^ = 0.007, df = 1, *p* = 0.931, >0.05) (Figure 6A_2_). The concentration of NT-3 in the culture supernatant was shown in Figure 7. The average purity of OEC in each group is shown in Table 1. (Supplemental Table S1).

### 2.2. Cell Viability

The MTT assay was used to measure cell viability. When the serum concentrations were the same, the OD values of Group A were higher than the values in Group B (Figure 6B_1_). Statistical differences were detected when comparing OD value of 5% serum with OD value three kinds of serum (concentration: 10%, 15%, 20%) (χ^2^ = (20.079, 24.806, 14.555), df = 1, *p* < 0.01), respectively (Figure 6B_1_). No statistically significant difference was observed for the different orders in which the serum was applied (χ^2^ = 0.446, df = 1, *p* = 0.054, >0.05) (Figure 6B_2_; Supplemental Table S2).

### 2.3. NT-3 Concentrations in the Supernatant

A statistically significant difference in the concentration of NT-3 was found when comparing NT-3 concentration of 5% serum with the 10% serum (χ^2^ = 7.491, df = 1, *p* = 0.011, <0.05) or 15% (χ^2^ = 6.524, df = 1, *p* = 0.006, <0.01) (Figure 6C_1_). OEC cultured in medium with RS secreted less NT-3 than OEC cultured in medium with FBS, and this difference was statistically significant (χ^2^ = 242.997, df = 1, *p* < 0.01) (Figure 6C_2_). Moreover, OEC cultured with spinal cord explants secreted less NT-3 than the cells cultured alone, and the difference was statistically significant (*F* = 9.611, df = 5.139, *p* < 0.01) comparing with the NT-3 concentration in 10% FBS (Figure 7). In addition, no statistically significant difference was observed for the different orders in which the serum was applied (χ^2^ = 0.148, df = 1, *p* (=0.700) > 0.05) (Figure 6C_1_,C_2_)) (Supplemental Table S3).

### 2.4. Real-Time Quantitative RT-PCR

**Figure 8:**
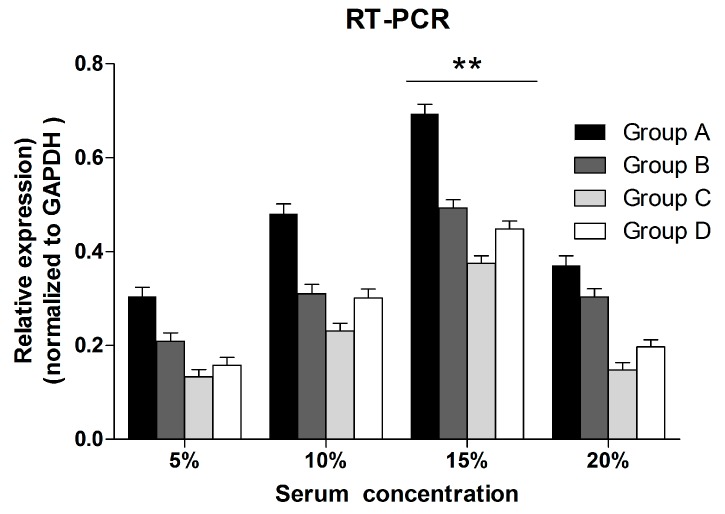
Quantitative RT-PCR assay was used to investigate the effect of different sera conditions on the expression of *NT-3* genes in OEC. The *NT-3* mRNA relative expression (normalized to GAPDH) of OEC in 15% serum was significantly different comparing with the OEC in 5% serum. The data was presented as mean ± SD. Two asterisks mean that there is statistically significant difference and *p* < 0.01.

The real-time quantitative RT-PCR was used to investigate the NT-3 gene expression in OEC. There was statistically significant difference among different groups when the serum concentration was 15% (*p* < 0.01). No statistically significant difference was found among groups when the serum concentration was 5%, 10% or 20% (Figure 8).

**Table 1:** OEC purity in each group.

Group	Purity of OEC
5% FBS	93.82%
10% FBS	95.83%
15% FBS	95.75%
20% FBS	98.49%
5% RS	96.56%
10% RS	96.61%
15% RS	96.18%
20% RS	95.01%
10% FBS–5% RS *	89.67%
10% FBS–15% RS *	95.33%
10% FBS–20% RS *	96.49%
5% FBS–5% RS *	94.25%
10% FBS–10% RS *	92.84%
15% FBS–15% RS *	90.88%
20% FBS–20% RS *	94.96%
10%FBS—10%RS + spinal cord explants **	96.98%

* OEC in this group were first cultured in DF-12 medium containing different concentration of FBS for 3 days and then cultured in DF-12 medium containing different concentration of RS for 4 days; ** OEC in this group were first cultured in DF-12 medium containing 10% FBS for 3 days and then cultured in DF-12 medium containing 10% RS and spinal cord explants for 4 days.

## 3. Discussion

SCI is a worldwide problem, and various therapies are utilized. Cell transplantation is a feasible alternative method. Various cell types such as neural stem cells [15], embryonic stem cells [16], mesenchymal stem cells [17,18], Schwann cells [19], mononuclear cells [20] and OEC [2,21] are currently being studied. Among these cell types, OEC have been applied for clinical use. Although OEC transplantation has been shown to be feasible and safe in humans [5,6,10,21,22], the curative effect is highly variable. Several studies suggest that the transplanted OEC remarkably improve motor function [23,24], and the effects are evident even in animals with complete spinal cord transection [24,25]. In contrast, other studies suggest that no functional improvement occurs after OEC transplantation [26,27]. Centenaro and colleagues postulated that this result is due to the insufficient axonal regrowth of the descending myelinated fibers at the lesion site [21].

Based on the fact that the survival condition of the OEC changed from the laboratory culture environment to the internal environment of the human body [10,11], we presumed that when the OEC became accustomed to the culture environment* in vitro*, transplantation may shock the cells, and this change may cause the curative effects of OEC transplantation to vary [7,8]. Considering that FBS is generally used in cell culturing in the lab and is commonly used for OEC culturing in most studies [10,11], we compared the influences of different concentrations of FBS and RS on OEC* in vitro*. RS, which was of the same genus as the OEC used in our research, was used to mimic the environment after transplantation. Moreover, this variance enabled us to study the change in OEC counts, viability, and NT-3 secretion after transplantation* in vitro*.

According to our statistical analysis, the cell counts peaked at the 10% and 15% serum concentrations, and the OD values for the MTT assay peaked at 15% serum; however, the difference in the NT-3 concentrations was not statistically significant. Considering the limited space in the 24-well format, when the cell density increases, the single cell volume decreases. This change may explain why the nuclei in the 5% concentration groups were bigger than the nuclei in the other three groups in the p75NTR immunofluorescence staining images (Figure 1, Figure 2, Figure 3 and Figure 4). Although, in theory, a higher concentration of serum leads to increased cell proliferation, contact inhibition limits proliferation [28]. Moreover, this inhibition explains why the OD value increased with the serum concentration, while a decrease was observed at a concentration of 20% serum (Figure 6B_1_,B_2_). The OD values in the MTT assay represent the viability of all living cells, including the OEC and other cells, but the OEC counts decreased in the 20% groups (Figure 6A_1_,A_2_).

Considering the results of our research, RS did not enhance OEC proliferation and NT-3 secretion compared with FBS. The key to this result was not the sequence of the different types of serum but the type of serum applied in the medium. When the OEC were acclimated to the environment* in vitro*, the transition to the* in vivo*-like environment may shock the cells, resulting in unsatisfactory cell proliferation and function. Combined with the comparison of OEC cultured with spinal cord explants and the cells cultured alone, this shock may explain why OEC are less healthy after transplantation than* in vitro*. In addition, changes in cell survival may also explain these results. However, in previous studies by Woodhouse* et al.*, the proliferation of ensheathing cells was significantly increased when co-cultured with explants from uninjured spinal cord [29]. Our results were different with Woodhouse’s due to two main reasons: first, we had serum alternation from FBS to RS; second, there were marked differences in the times in culture and subculture. Other factors, such as different species of animals, different methods for primary culture and purification, as well as different supplements added to the medium also contributed to the varies.

**Figure 9:**
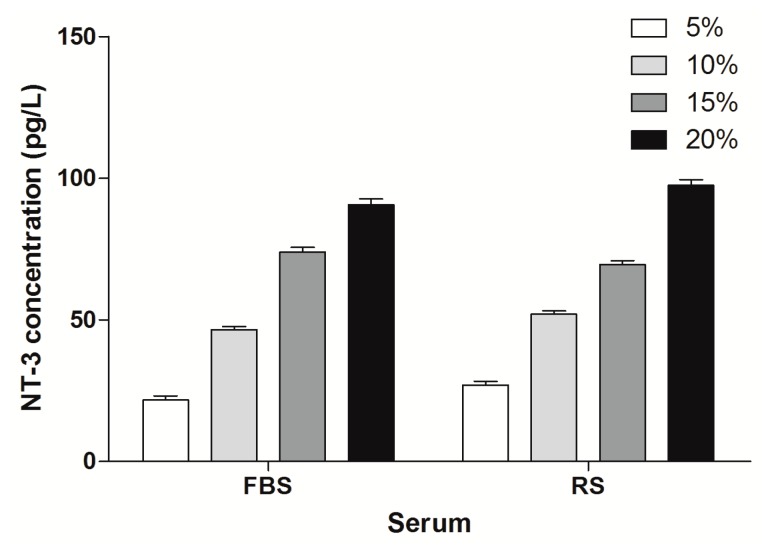
The concentration of NT-3 in the culture medium containing different concentrations of serum was detected by ELISA. There are eight kinds of medium: (1) DMEM/F12 with 5% fetal FBS; (2) DMEM/F12 with 10% FBS; (3) DMEM/F12 with 15% FBS; (4) DMEM/F12 with 20% FBS; (5) DMEM/F12 with 5% RS collected from adult male S-D rats; (6) DMEM/F12 with 10% RS; (7) DMEM/F12 with 15% RS; and (8) DMEM/F12 with 20% RS. The data was presented as mean ± SD.

Considering that there is a low level of NT-3 in the blank control of serum (Figure 9), the RT-PCR was used to investigate the *NT-3* gene expression in OEC. It is observed that when the serum concentration was 15%, the *NT-3* gene expression in Group A was higher than other groups, in which RS is applied (Figure 8). Thus, it could be concluded that the switch of sera condition has a negative effect on the *NT-3* gene expression in OEC* in vitro*.

The limitations of this study must be acknowledged. Although we aimed to simulate the growing environment of OEC after transplantation, this study focused on the serum species and four different serum concentrations. The actual environment is much more complicated. Therefore, further* in vivo* studies should be conducted to transplant cultured OEC cells into the rat spinal cord and to perform dynamic monitoring on these cells.

## 4. Materials and Methods

### 4.1. Experimental Animals

Seventy adult male Sprague–Dawley (S–D; 167.82 ± 6.95 g, 150–180 g) rats were used to collect rat serum (RS), and fifteen 3-week-old S–D rats were used to isolate olfactory bulbs. Twelve adult S–D rats were used for spinal cord explants. All studies were performed with the approval of and under the guidelines of the Animal Care Committee of Xi’an Jiaotong University.

### 4.2. Culture Medium Preparation

**Figure 10:**
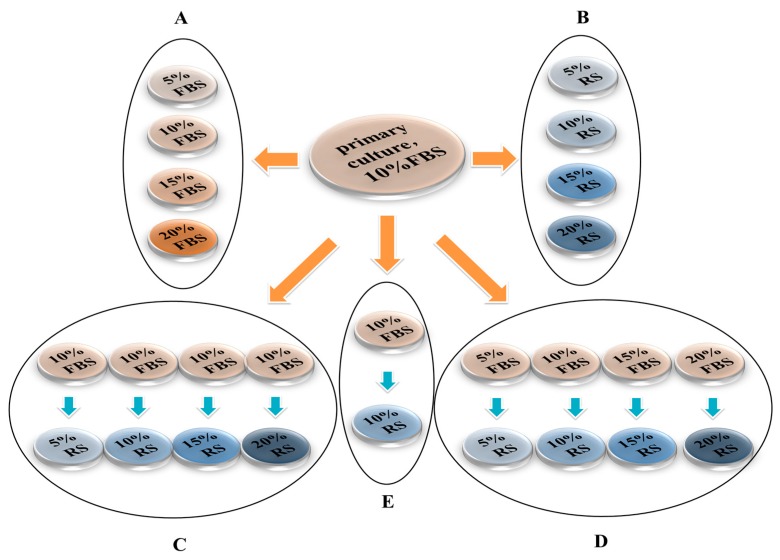
Illustration of the experimental groups in this study. Eight kinds of medium contained 5%, 10%, 15% or 20% FBS or RS. OEC were first cultured in 10% FBS to complete the primary culture. The OEC subculture was divided into 5 treatment groups as follows: (**A**) after trypsinization, OEC were placed onto slides and cultured in different concentrations (5%, 10%, 15%, 20%) of FBS for 7 days; (**B**) after trypsinization, OEC were cultured in different concentrations (5%, 10%, 15%, 20%) of RS for 7 days; (**C**) after trypsinization, OEC were cultured in 10% FBS for 3 days and then cultured in four different concentrations (5%, 10%, 15%, 20%) of RS for another 4 days; (**D**) after trypsinization, OEC were placed onto slides, cultured in four different concentrations (5%, 10%, 15%, 20%) of FBS for 3 days, and then cultured in RS (concentrations: 5%, 10%, 15%, 20%) for another 4 days; and (**E**) the OEC were first cultured in 10% FBS for 3 days and then cultured with rat spinal cord explants with 10% RS for another 4 days. The orange arrows indicate that trypsin was applied, and the blue arrows indicate that trypsin was not applied.

Eight types of culture media containing different concentrations and types of serum were used: (1) Dulbecco’s modified Eagle medium supplemented with F12 (DMEM/F12, Gibco, Carlsbad, CA, USA) and 5% fetal bovine serum (FBS) (Gibco); (2) DMEM/F12 with 10% FBS; (3) DMEM/F12 with 15% FBS; (4) DMEM/F12 with 20% FBS; (5) DMEM/F12 with 5% RS collected from adult male S–D rats; (6) DMEM/F12 with 10% RS; (7) DMEM/F12 with 15% RS; and (8) DMEM/F12 with 20% RS (Figure 10).

### 4.3. Spinal Corsd Explants Preparation

The spinal cord explants was extracted from adult male S–D rats. The rats were deeply anesthetized with 10% chloral hydrate (Sigma Chemical Company, St. Louis, MO, USA) and then were placed on the operation platform. A skin incision was made in the back, and the spinous process was resected. Then the spinal cord spanning the T8–T12 vertebrae was dissected free of nerve roots and placed in cold Hank’s balanced salt solution (HBSS, Invitrogen, Carlsbad, CA, USA). The meninges and blood vessels on the spinal cord were divested with forceps under the microscope after harvesting. The spinal cord was cut into small pieces (0.5 mm^3^) and digested with 0.125% trypsin (Sigma) for 20 min at 37 °C and 5% CO_2_. DF-12 medium (1:1 mixture of DMEM and Ham’s F-12) containing 10% fetal bovine serum (FBS, Gibco) was used to terminate the digestion. After centrifugation at 1000 rpm/min for 5 min, the pellet was resuspended in the same medium described above. The spinal cord explant of one segment was added to each culture well and co-cultured with OEC in the subculture in a humidified atmosphere containing 5% CO_2_ at 37 °C.

### 4.4. Primary Culture and OEC Purification

Following the procedures of Nash* et al.* [11], olfactory bulbs were removed from the heads of 3-week-old rats. The rats were deeply anesthetized with 10% chloral hydrate (Sigma Chemical Company, St. Louis, MO, USA) and decapitated. A skin incision was made in the scalp, and the occipital bone of the skull was resected. Then, the olfactory bulbs were removed with microforceps and placed in cold Hank’s balanced salt solution (HBSS, Invitrogen, Carlsbad, CA, USA). HBSS was maintained on ice during harvesting. The meninges and blood vessels on the olfactory bulbs were divested with forceps under the microscope after harvesting. The olfactory nerve and glomerular layers were dissected and retained. The tissue was cut into small pieces and then digested with 0.125% trypsin (Sigma) for 20 min at 37 °C and 5% CO_2_. DF-12 medium (1:1 mixture of DMEM and Ham’s F-12) containing 10% fetal bovine serum (FBS, Gibco) was used to terminate the digestion. After centrifugation at 1000 rpm/min for 5 min, the pellet was resuspended in the same medium described above. The cells were seeded into culture flasks (25 cm^2^, Nunclon, Thermo Scientific, Waltham, MA, USA) and incubated in a humidified atmosphere containing 5% CO_2_ at 37 °C. The differential adherence method described by Nash* et al.* was used to purify the primary cells [11]. After purification, the medium was changed every two days.

### 4.5. OEC Subculture

After purification and primary culturing for 7 days, all cultures were trypsinized to remove the cells from the flasks, and the cells were counted using a hemocytometer. Then, the OEC were resuspended in DF-12 for the uses below. Next, the cells were placed onto slides and were cultured in different media. The OEC in Group A and Group B were cultured in four different serum concentrations (5%, 10%, 15%, 20%) for 7 days (Figure 10A,B). Simultaneously, the trypsinized OEC were placed onto slides and cultured in four different concentrations (5%, 10%, 15%, 20%) of FBS for 3 days, and then the medium was removed with a pipette. Next, without trypsinization, these cells were cultured in four different concentrations of RS (Figure 10C,D) for 4 days. In group E, the OEC were first cultured in 10% FBS for 3 days and then cultured with rat spinal cord explants with 10% RS for another 4 days (Figure 10E).

### 4.6. MTT Assay

The cell suspension was diluted to 2.0 × 10^5^ cells/mL, and the cells were seeded onto 96-well plates at 10 μL per well. Then, the seeded cells were cultured for 7 days using different media according to the different groups (Figure 10). After 7 days, the MTT (methyl thiazolyl tetrazolium) assay was performed to detect cell viability. Due to the existence of a large number of unknown cells from rat spinal cord explants, the MTT assay was not performed in this group.

### 4.7. Neurotrophin-3 (NT-3) Concentration Measurements 

The cell suspension was diluted to 1.0 × 10^5^ cells/mL, and the cells were seeded onto coverslips that were pre-coated with poly-l-lysine and placed at the bottom of 24-well plates at a volume of 30 μL per well. Then, the cells were cultured for 7 days using different media according to the different experimental groups (Figure 10).

On the 7th day, the supernatant was collected to measure the NT-3 concentration using an ELISA kit (CUSABIO, Wuhan, China). The ELISA procedure was as follows. All reagents and samples were brought to room temperature before use. The samples were centrifuged again before the assay, and 100 μL of standard and sample was added to each well. The plate was covered with an adhesive strip and incubated for 2 h at 37 °C. Then, the liquid in each well was removed, and 100 μL of biotin antibody (1×) was added to each well. The plate was covered with a new adhesive strip and incubated for 1 h at 37 °C. Next, each well was aspirated and washed, and this process was repeated twice for a total of three washes. Each well was washed with 200 μL of wash buffer, and each wash lasted for 2 min before the complete removal of liquid at each step. After the last wash, the plate was inverted and blotted with clean paper towels. Then, 100 μL of HRP–avidin was added to each well, and the microplate was covered with a new adhesive strip and incubated for 1 h at 37 °C. The wash process described above was repeated five times after incubation. Next, 90 μL of TMB substrate was added to each well, and the plate was incubated for 15–30 min at 37 °C protected from light. Finally, 50 μL of stop solution was added to terminate the reaction. The optical density of each well was determined within 5 min using a microplate reader set to 450 nm.

### 4.8. Immunofluorescence Staining

The OEC were rinsed three times in phosphate-buffered saline (PBS) before being fixed in 4% PFA for 20 min, and then the cells were rinsed another three times in PBS. For immunofluorescence, the cells were pre-incubated with 10% goat serum albumin (Sigma) for 60 min at room temperature and rinsed three times in PBS, followed by a 12 h incubation at 4 °C with a rabbit anti-rat P75NTR monoclonal antibody (1:50, Proteintech, Chicago, IL, USA). The secondary goat anti-rabbit antibodies Cy3 (Proteintech) were used at 1:500, and the cells were incubated for 60 min at room temperature. At the 55th min, DAPI (100 ng/mL) was added. Then, the cells were rinsed three times in PBS before being mounted in 50% glycerol. Under 200× magnification, 5 different microscopic fields (200×) were randomly chosen. The number of OEC in each field was calculated and summed to obtain an average number of OEC in each group.

### 4.9. Real-Time Quantitative RT-PCR

The gene expression levels of NT-3 and glyceraldehyde-3-phosphate dehydrogenase (GAPDH) (as an Internal control) genes in cells with varying treatments were determined by RT-PCR. Total cellular RNA was extracted from the OEC after subculture using RNAiso Plus (Takara Inc., Dalian, China) according to the instructions of the manufacturer. Then complementary DNA (cDNA) was synthesized from 1 μg of total RNA with reverse transcriptase with random primers and PrimeScript RT reagent Kit (Takara Inc., Dalian, China) for quantitative RT-PCR. The process of RT-PCR was performed on a Thermal Cycler Dice Real Time System (Takara Inc., Dalian, China) using SYBR Premix Ex Taq™ (Takara Inc., Dalian, China) according to the instructions of manufacturer. The reaction mixture was subjected to PCR amplification for 40 cycles consisting in heat denaturation, annealing and extension. Each sample was run in triplicate. To detect NT-3 transcripts we used: 5'-CAAACCTCCAAAGTGCTGTGT-3' (forward) and 5'-GGGGTGAATTGTAGCGTCTCT-3' (reverse). To detect GAPDH transcripts we used: 5'-TTCAACGGCACAGTCAAGG-3' (forward) and 5'-CATGGACTGTGGTCATGAG-3' (reverse). The RT-PCR was not applied in the group in which OEC were co-cultured with spinal cord explants because the OEC and the explants cannot be separated.

### 4.10. Image Analysis and Statistics

Digital images were captured with an inverted fluorescence microscope (Nikon Ti-E, Tokyo, Japan) and were analyzed with Ismage-Pro Plus 6.0 software (Media Cybernetics, Rockville, MD, USA). In this study, the data were analyzed using SPSS software (version 13.0; SPSS Inc., Chicago, IL, USA). The OEC count, MTT test, NT-3 concentration and qRT-PCR results were analyzed using the multivariate analysis of a generalized linear model. Four different kinds of serum concentration (5%, 10%, 15%, 20%), two kinds of serum type (FBS and RS) and three processes in subculture (from FBS to FBS, from RS to RS, from FBS to RS) were considered as variables. OEC number, OD value and NT-3 concentration were considered as dependent variables. Considering 5% was the initial concentration, 5% serum was taken as a base line when comparing the differences between groups. The results of the group in which OEC were cultured with spinal cord explants were analyzed using a *t*-test. *p* values less than 0.05 were considered statistically significant. All analyses were conducted by a researcher blinded to the treatment groups.

## 5. Conclusions

The OEC counts, cell viability and NT-3 concentration were all influenced by the different types of serum used during the culturing process. FBS was more conducive to OEC proliferation compared with RS* in vitro*; however, the order in which the different sera were applied did not result in statistically significant differences. Thus, the results indirectly suggest that the switch in the extracellular environment may lead to a change in the quantity and quality of the cells, which may be responsible for the unsatisfactory curative effects of OEC transplantation. Further studies are necessary to determine the exact factors that inhibit OEC* in vivo*.

## Supplementary Materials

Supplementary materials can be found at http://www.mdpi.com/1422-0067/16/01/0420/s1.
